# Remarkable Changes in Behavior and Physiology of Laboratory Mice after the Massive 2011 Tohoku Earthquake in Japan

**DOI:** 10.1371/journal.pone.0044475

**Published:** 2012-09-05

**Authors:** Shuichi Yanai, Yuki Semba, Shogo Endo

**Affiliations:** Aging Regulation Research Group, Tokyo Metropolitan Institute of Gerontology, Itabashi, Tokyo, Japan; University of Houston, United States of America

## Abstract

A devastating earthquake and tsunami hit Japan on March 11, 2011, followed by several long and intense aftershocks. Laboratory mice housed in the Tokyo, located approximately 330 km south of this earthquake’s epicenter, displayed remarkable changes in a variety of behaviors and physiological measures. Although unusual pre-earthquake behaviors have been previously reported in laboratory animals, little is known about behavioral and physiological changes that occur after a great earthquake. In the present study, the effects of Tohoku earthquake on mice behavior were investigated. “Earthquake-experienced” mice displayed a marked increase in food consumption without gaining body weight in response to the earthquake. They also displayed enhanced anxiety, and in a formal fear memory task, showed significantly greater tone- and context-dependent conditioned freezing. Water maze performance of earthquake-experienced mice showed the quicker acquisition of the task, faster swim speed and longer swim distance than the naive mice. Serum corticosterone levels were elevated compared to the naive mice, indicating that the earthquake and aftershocks were stressful for the mice. These results demonstrate that great earthquakes strongly affect mouse behaviors and physiology. Although the effects of a variety of experimental manipulations on mouse behaviors in disease models or in models of higher cognitive functions have been extensively examined, researchers need to be aware how natural phenomena, such as earthquakes and perhaps other natural environmental factors, influence laboratory animal behaviors and physiology.

## Introduction

Rodents are the most widely used animal research subjects, comprising about 95% of all laboratory animals used in scientific research [1]. The value of mouse subjects is especially great due to the availability of a wide range of genetically modified mice and due to the ability to easily control experimental manipulations and conditions for these subjects. Transgenic or knockout mouse models have proven to be useful in understanding normal cognitive processes [2], and cognitive impairments in diseases such as Alzheimer’s disease [3]. Mice behaviors are, however, often affected by unexpected and/or uncontrollable external factors, in addition to intended experimental manipulations.

Earthquakes are unavoidable natural disasters that are not only a significant threat to humans but also to animals in nature. Although humans are unable to detect seismic precursors or to reliably predict their occurrence, several studies have reported that many animals display unusual, apparently prescient, pre-earthquake behaviors [4]–[7]. For example, unusual mouse behaviors were observed before the Kobe earthquake of January 17, 1995 [7]. Mouse locomotor activity increased drastically during both nocturnal and diurnal circadian phases one day before the earthquake [7]. Among the several kinds of geophysical variations that occur before earthquakes, the altered magnetic fields precursor may account for abnormal behaviors in laboratory mice [5]. In spite of such pre-earthquake studies, little is known about the behavioral changes that occur after earthquakes. Previous research has demonstrated that earthquakes increase the prevalence of psychiatric distresses such as depression and anxiety in humans [8]. Taken together, these results led us to hypothesize that earthquakes may also influence an aspect of behaviors in laboratory animals.

On March 11, 2011, a historic great earthquake, followed by a devastating tsunami, hit Japan. This earthquake registered a magnitude 9.0 on the Richter scale. The central and northwest part of the main island of Japan continued to experience numerous aftershocks for months. Tokyo, located approximately 330 km south of the epicenter, was hit by an earthquake measuring a maximum intensity 5-upper on the Japanese Meteorological Agency (JMA) seismic intensity scale (comparable to level VII on the Modified Mercalli intensity scale). These two scales measure the destructive effects of an earthquake at any given point on the earth, whereas the Richter scale measures the energy released by an earthquake.

Before the earthquake, our laboratory at Tokyo was housing wild-type C57BL/6 mice intended for subsequent research. These mice experienced not only the major seismic event but also several aftershocks. These “earthquake-experienced mice” exhibited a remarkable increase in food consumption immediately following the main earthquake. For over a month, their food consumption continued to be significantly higher than that of mice not exposed to the earthquake. This unusual feeding behavior prompted us to investigate in detail how earthquakes influence other behaviors in mice and their physiology.

## Materials and Methods

### 1 Ethics Statement

All experiments were approved by the animal experiment committee of the Tokyo Metropolitan Institute of Gerontology and carried out according to its guidelines.

### 2 Apparatus

Apparatus for open field test, fear conditioning task, Morris water maze task, and hot plate test were obtained from O’Hara & Co., Ltd. (Tokyo). Automated software Time for Morris water maze and fear conditioning (O’Hara & Co., Ltd, Tokyo) or Image OFCR for open field (O’Hara & Co., Ltd, Tokyo) were utilized to control experimental devices and to analyze the obtained data. The Image OCFR is the software based on NIH Image (developed at the U.S. National Institute of Health).

### 3 Subjects

Experimentally naive male C57BL/6J mice were obtained from CLEA Japan, Inc. (Tokyo). Mice were housed in groups of four or five per cage with wood chip bedding. Rodent feed (CRF-1, Oriental Yeast, Ltd, Tokyo) and wood chip bedding (Oriental Yeast, Ltd, Tokyo) were autoclaved at 120°C for 2 h before use. The drinking tap water (supplied by the Bureau of Waterworks, Tokyo Metropolitan Government) was filtered and its chlorine concentration was adjusted to12±2 ppm with pH of 2.5–3.0 by adding sodium hypochlorite and hydrochloric acid, then, was provided as drinking water for mice. The vivarium was maintained at 24±1°C with a 12 hour-12 hour light-dark cycle (light on at 7∶00 AM). “Earthquake-experienced mice” (n = 47) were defined as mice that experienced the March 11th earthquake and subsequent aftershocks. Behavioral experiments for earthquake-experienced mice were conducted between April 4 and 30 (25 to 51 days after the main seismic event). “Naive mice” (n = 46) are mice that were subjected to experiments *before* March 10 (i.e., these did not experience the main earthquake and aftershocks). These latter mice were used in our research between November 28, 2010 and February 11, 2011. Behavioral experiments were conducted when they were between 11 and 20 weeks of age.

### 4 Procedure

After mice were handled approximately 5 min per day for 3 consecutive days, they were tested in behavioral experiments. All behavioral experiments were conducted between 9∶00 AM and 17∶00 PM.

#### 4–1. Measurement of food consumption and body weight

Twenty-four earthquake-experienced mice were in the course of another experiment when the main seismic event hit them. They were fed *ad libitum* for 4 hours from 10∶00 AM to 2∶00 PM. They had free access to drinking water in their cage at all times. Their daily food consumption was measured by weighing the food and calculated over 4-hr free feeding period. Larger pieces of spilled food was gathered up and replaced in the food container for weighing. Since food consumption was measured for each cage, mean food consumption per cage was regarded as representative value for 4 mice housed in one cage. The measurement of food consumption on March 11 was completed prior to the main seismic event.

#### 4–2. Morris water maze task

Twelve from 24 earthquake-experienced mice whose daily food consumption had been observed, and 6 from 24 naive mice were tested in the Morris water maze to examine spatial memory [9]. A standard training protocol was used [10]. Briefly, mice were allowed to swim (60 s maximum) to the submerged escape platform that was placed at a fixed position through an acquisition period. Four trials were conducted each day until their performance reached asymptote. On the next day at the completion of acquisition training, a probe test was conducted for 60 s with the platform removed.

#### 4–3. Behavioral test battery

Twenty-three earthquake-experienced and 22 naive mice were sequentially subjected to open field test, Pavlovian fear conditioning task, and hotplate test. The order of testing was determined according to the observation that preceding task experience did not affect succeeding task performance [11]. In the Pavlovian fear conditioning task, mice were trained either by weak training protocol or by strong training protocol (see below). Those mice used in the behavioral test battery were not in food restriction nor trained in the Morris water maze. These mice had free access to both food and drinking water during the experiments. The basic procedures for the behavioral experiments were described previously [12].

##### Open field test

Spontaneous motor activity, exploratory behavior, and emotional responses in a novel environment were automatically measured. Briefly, a mouse was placed in the open field (50×50×50 cm) and allowed to explore for 15 min. Performance was assessed under dark-lit conditions (28 lx) on the first day, followed by bright-lit conditions (150 lx) on the next day. Distance traveled, number of rearing, time spent in the center of the field, and immobile time were measured.

##### Pavlovian fear conditioning task

Conditioned fear to tone and context was measured. After conditioning with the conditioned stimulus (CS; 10 kHz, 70 dB tone for 3 s in both training protocol) and the unconditioned stimulus (US; 0.5 s electrical footshock, 0.12 mA for weak and 0.30 mA for strong training protocols), mice were sequentially examined for short-term (1 h) and long-term (24 h) tone-dependent fear memory, followed by context-dependent fear memory (48 h). For the non-conditioned naive mice, CS was presented without US. Throughout the experiments, freezing was used as an index of fear [13].

#### Hotplate test

Pain sensitivity was assessed by the latency to lick a paw after mice was placed on a 55°C hotplate.

#### Serum collection and corticosterone measurement

Three weeks after the completion of behavioral test battery, 8 earthquake-experienced and 11 naive mice, not in food restriction, were deeply anesthetized by isoflurane in their home cage. Then, trunk blood was collected without decapitation. Blood collection was conducted between 16∶30 and 17∶00. Blood samples were allowed to clot for 30 min before centrifuging for 15 min at 3000×g. Serum was then collected and subjected to corticosterone assay using an ELISA kit (Assay Pro, St. Charles, MO) according to the manufacturer’s instructions.

## Results

**Figure 1:**
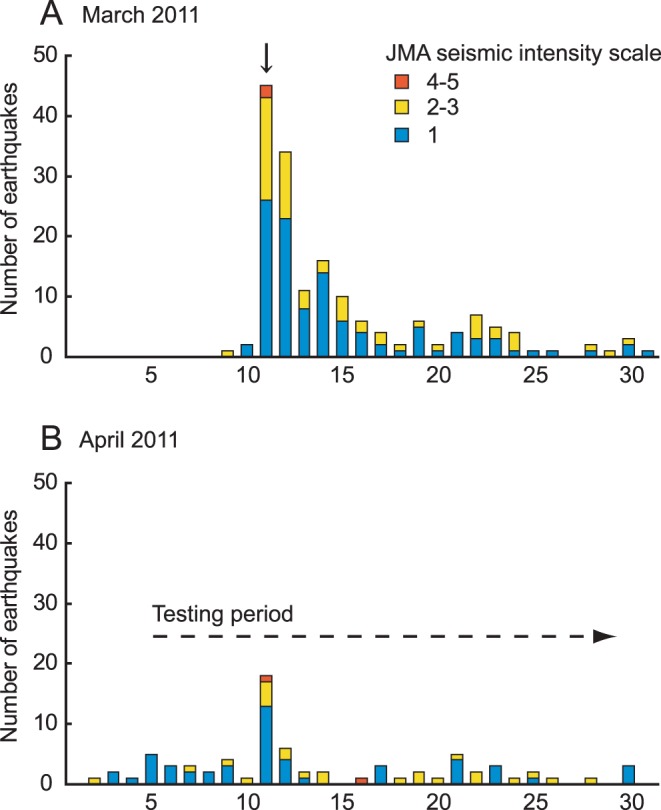
Number of earthquakes and its intensity observed in the Tokyo area. (A) Daily occurrence of earthquakes and its intensity observed in March, 2011. A major seismic event of the Tohoku earthquake occurred on March 11, 2011 (black arrow). On the day of the main seismic event, total number of 45 earthquakes were observed in the Tokyo area. (B) Daily occurrence of earthquakes and its intensity observed in April, 2011. Behavioral experiments for the earthquake-experienced mice were conducted between April 4 and 30 (dotted arrow). Classification of earthquake were according to the JMA seismic intensity scale.

A major seismic event measuring intensity 5-upper on the JMA seismic intensity scale occurred on March 11, 2011, followed by numerous aftershocks (Figure 1A; data relating to the earthquake were obtained from Japan Meteorological Agency). A few much smaller earthquakes were recorded in the Tokyo area before March 10. Although the number of aftershocks decreased dramatically in a few days, repeated aftershocks still continued during the testing period for the earthquake-experienced mice (Figure 1B). By contrast, very few earthquakes were recorded during the testing period for the naive mice (Table 1). On the day of the major seismic event, the air handling unit of the vivarium was down upon the event. The temperature of animal housing room decreased by approximately 5°C for 3 hours. The air handling unit was repaired immediately and the room temperature went back to normal in 1 hour and became stable thereafter. No other failures to disturb vivarium environment were observed. Further, a large stock of rodent feed, bedding and clean cages made us possible to continue the routine care of mice after the earthquake. Alteration of dissolved organic compounds in the water has been considered to function as the seismic precursor in the wild animals [4], however, apparent changes were not detected in chemical component of water sampled at three time points from October 2010 to May 2011 (Table 2; data relating to the water chemistry were obtained from the Bureau of Waterworks, Tokyo Metropolitan Government) to cover the data for before or after the earthquake.

**Table 1:** Number of earthquake and its intensity observed in the Tokyo area during the testing period for naive mice. *

			JMA seismic intensity scale	
Year	Month	Day	1	2–3
2010	November	30		1
	December	6		1
		11	1	
		22		1
2011	January	8	1	

* The data relating to the earthquake were obtained from Japan Meteorological Agency. The earthquake greater than intensity scale 2–3 was not observed in this period.

**Table 2:** Water chemistry data in the Tokyo area. *

Chemical component	Standard value**	2010 Oct 13	2011 March 9	May 12
Heavy metals and ions				
Aluminium	0.2 mg/ml	0.02	0.02	0.02
Chlorate	0.6 mg/ml	0.03	0.02	0.00
Chloride ion	200 mg/ml	20.1	27.0	17.5
Chromium (VI)	0.05 mg/ml	0.000	0.000	0.000
Copper	1.0 mg/ml	0.00	0.00	0.00
Cyanide ion	0.01 mg/ml	0.000	0.000	0.000
Iron	0.3 mg/ml	0.00	0.00	0.00
Lead	0.01 mg/ml	0.000	0.000	0.000
Manganese	0.05 mg/ml	0.000	0.000	0.000
Zinc	1.0 mg/ml	0.00	0.00	0.00
Organic compounds				
Total organic carbon	3 mg/ml	0.6	0.7	0.6
1,4-dioxane	0.05 mg/ml	0.0000	0.0000	0.0000
Benzene	0.01 mg/ml	0.0000	0.0000	0.0000
Bromate	0.01 mg/ml	0.001	0.000	0.000
Bromodichloromethane	0.03 mg/ml	0.0049	0.0047	0.0036
Bromoform	0.09 mg/ml	0.0017	0.0014	0.0012
Carbon tetrachloride	0.002 mg/ml	0.0000	0.0000	0.0000
Chloroacetic acid	0.02 mg/ml	0.000	0.000	0.000
Chloroform	0.06 mg/ml	0.0029	0.0034	0.0021
1,2-Dichloroethylene	0.04 mg/ml	0.0000	0.0000	0.0000
Dibromochloromethane	0.1 mg/ml	0.0058	0.0045	0.0040
Dichloroacetic acid	0.04 mg/ml	0.002	0.002	0.002
Dichloromethane	0.02 mg/ml	0.0000	0.0000	0.0000
Formaldehyde	0.08 mg/ml	0.001	0.002	0.001
Tetrachloroethylene	0.01 mg/ml	0.0000	0.0000	0.0000
Total trihalomethanes	0.1 mg/ml	0.015	0.014	0.011
Trichloroacetic acid	0.2 mg/ml	0.002	0.002	0.001
Trichloroethylene	0.03 mg/ml	0.0000	0.0000	0.0000
Bacteria				
Standard plate count	100 cfu/ml	0.0	0.0	0.0
Escherichia coli	nd	nd	nd	nd

* The water quality in Maeno-cho, Itabashi, Tokyo, the water sampling point close to our institute. Inspection data for the water quality is obtained from the Bureau of Waterworks, Tokyo Metropolitan Government who routinely monitors the quality of water supply.

** Standard values are for the water quality adequate for human consumption according to Japanese Law. cfu, colony-forming unit; nd, not detected.

**Figure 2:**
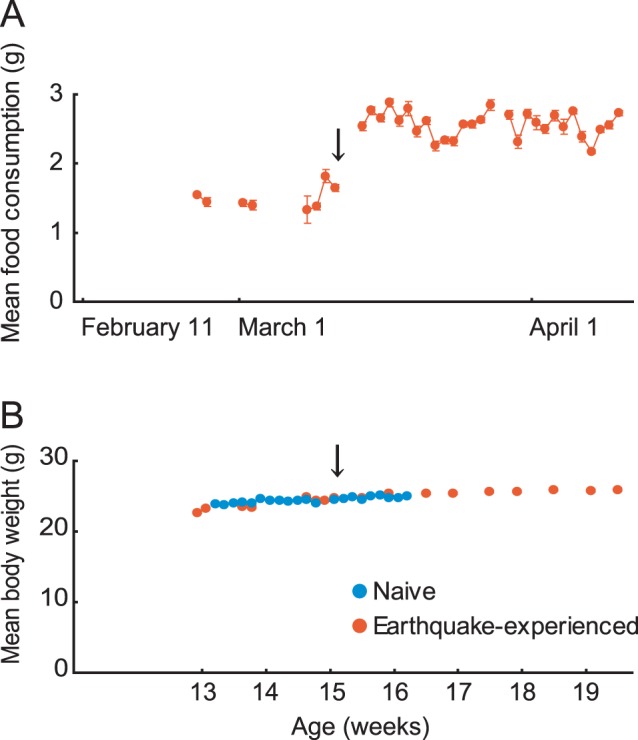
Number of mice food consumption and its body weight. (A) Mean food consumption of 24 earthquake-experienced mice significantly increased after the main seismic event. Since mice were housed in groups of four per cage, mean food consumption was calculated per cage and was regarded as representative value for four mice housed in one cage. Error bars indicate S.E.M. (B) Mean body weight of earthquake-experienced (n = 24) and age-matched naive mice (n = 24). Black arrows in both figures indicate March 11, 2011, when the main seismic event of the Tohoku earthquake occurred. Note that x-axis represents weeks of age and that the data for age-matched naive mice were obtained before March 11, 2011.

The first behavioral abnormality we noticed was a remarkable increase in food consumption immediately after the main earthquake (Figure 2A). Daily food consumption per mouse, under restricted feeding, was 1.7±0.13 (Mean±SD) g on March 11. The Tohoku earthquake occurred approximately 1 hour after the end of an *ad libitum* feeding period. On March 14, three days after the main seismic event, mean food consumption increased to 2.5±0.16 g (Figure 2A). Analysis using a paired t-test revealed that the increase in food consumption was significant after the earthquake (*t* (5) = 18.5, *p*<0.001). Even though the approximately 50% increase in food consumption continued for over one month, the body weight of earthquake-experienced mice increased only slightly (24.7±0.7 g on March 11 vs. 25.9±0.6 g on April 11), which was comparable to the weight gain seen in age-matched naive mice under the same feeding schedule (Figure 2B). No significant differences were observed in body weight between earthquake-experienced and naive mice (*t* (46) = 1.50, *n.s.*), measured at 16 weeks of age.

**Figure 3:**
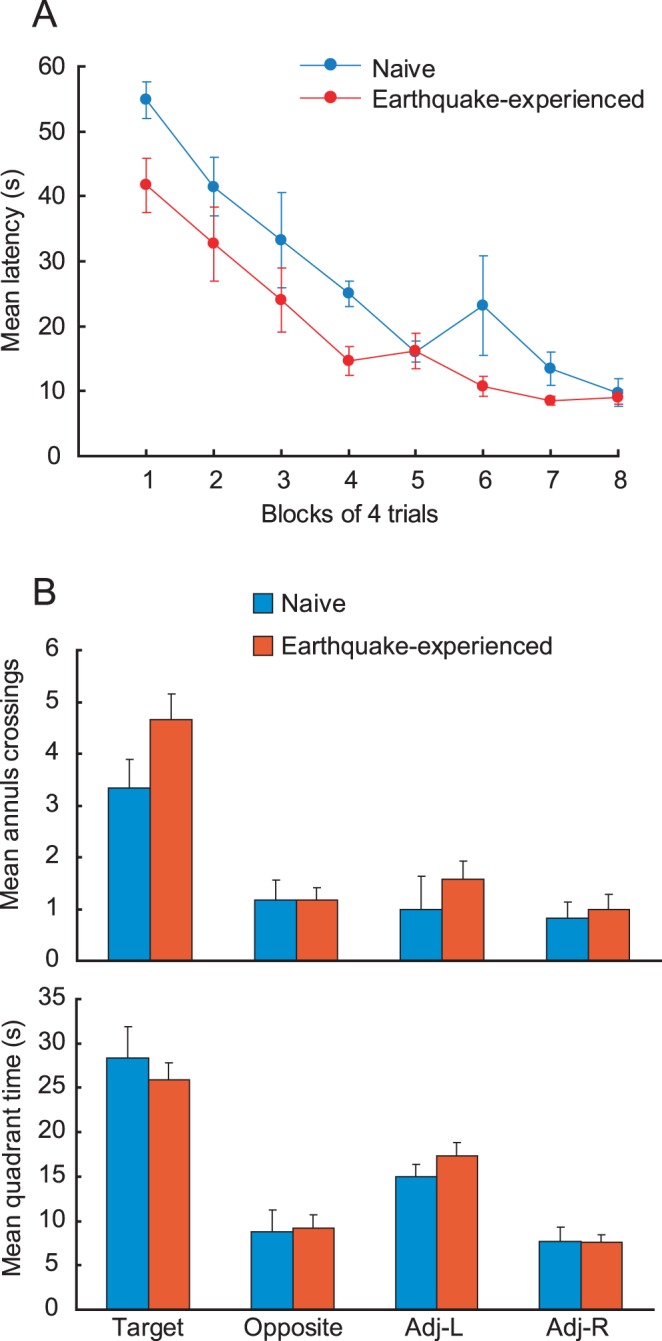
Spatial memory testing in the Morris water maze task. Earthquake-experienced (n = 12) and naive (n = 6) mice under a dietary restriction schedule were trained until their performance reached asymptote. Dietary regimen and its duration were the same for both groups of mice. (A) Mean escape latency to platform during acquisition training. Earthquake-experienced mice found the escape platform significantly faster than naive mice from an early stage of training. However, the asymptotic escape latency of both groups of mice was equivalent. (B) Mean annuls crossings (upper) and mean quadrant time (lower) in the probe test. The probe test was carried out on the day after the completion of an 8-day training period. There were no significant differences in two indices of the probe test. Error bars indicate S.E.M.

In the Morris water maze task, we observed a group difference in escape latency during the early stage of training. As training proceeded, this performance difference gradually diminished and asymptoted in 8 days (Figure 3A). A two-way ANOVA (with group as the between-subject factor and training blocks as the within-subject factor) revealed that the escape latency of earthquake-experienced mice was significantly shorter than that of naive mice (*F* (1, 16) = 7.73, *p*<0.05). A two-way ANOVA for the swim distance revealed that an interaction between groups and training blocks were significant (*F* (7, 112) = 2.26, *p*<.05) without significance in the main effect of group (*F* (1, 16) = 0.11, *n.s.*). The analysis of the simple main effect on training blocks revealed that the earthquake-experienced mice swan longer than naive mice only on day 1 (*p*<.05). Detailed analysis on day 1 revealed that the mean swim speed was 14.6±2.1 cm/s and 7.9±2.3 cm/s for earthquake-experienced and naive mice, respectively. A t-test revealed that earthquake-experienced mice swam faster than naive mice (*t* (16) = 6.09, *p* = 0.001). There were no significant differences in swim speed on day 8. Furthermore, no significant differences were observed between earthquake-experienced and naive mice in the two indices of the probe test carried out on the day after the completion of training (Figure 3B). These results indicate that the earthquake-experienced mice and the naive mice performed differently in the water maze, especially, in early stage of acquisition training.

In the open field test, significant group differences were found in distance traveled and immobile time on the first day (dark-lit condition). Distance traveled was 4616±715 cm for earthquake-experienced mice and 5436±941 cm for naive mice. A t-test revealed that the distance traveled by earthquake-experienced mice was significantly shorter than that by naive mice (*t* (43) = 3.30, *p*<0.01), indicating that earthquake-experienced mice had reduced locomotion. Immobile time was 42.6±2.9% for earthquake-experienced mice and 38.9±3.3% for naive mice, a significant increase revealed by t-test (*t* (43) = 3.92, *p*<0.001), indicating that the earthquake-experienced mice were in the state of higher anxiety. Significant negative correlation between distance traveled and immobile time were found for the earthquake-experienced (*r* = −0.97, *p*<.001) and the naive mice (*r* = −0.96, *p*<.001). Traditionally, immobile time and time spent in the center of the field are considered to reflect anxiety levels, and distance traveled is considered to reflect locomotor activity in the open field test [11], [14]. Since strong negative correlations were found between traveled distance and immobile time in our study, longer immobile time and shorter travel distance are also the index of higher anxiety [14]. No significant differences were found in number of rearing and time spent in the center of the field on the first day (dark-lit condition). No significant differences were found on the second day of testing (bright-lit condition).

**Figure 4:**
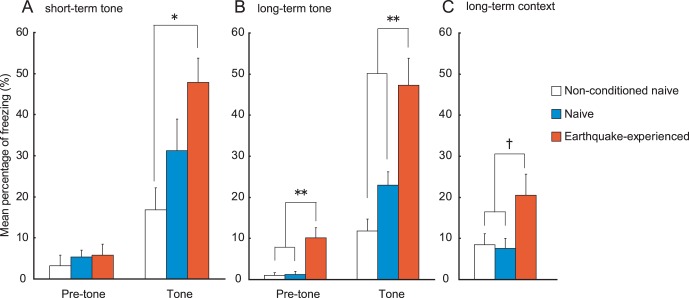
Pavlovian fear conditioning using a weak training protocol. After conditioning, conditioned freezing to tone and context was sequentially measured in earthquake-experienced (n = 10), naive (n = 7), and non-conditioned naive mice (n = 6). (A) Tone-dependent short-term memory test was carried out 1 h after the conditioning. No differences were found among the three groups of mice before the presentation of the tone. However, the earthquake-experienced mice exhibited significantly higher freezing than non-conditioned naive mice during tone presentation. (B) Tone-dependent long-term memory examined 24 h after the conditioning. Conditioned freezing of earthquake-experienced mice was significantly higher than that of the other two groups of naive mice before and during the tone. (C) Context-dependent long-term memory examined 48 h after the conditioning. Conditioned freezing to context was higher in earthquake-experienced mice than in the other two groups of naive mice. †*p*<0.10, *p<0.05, **p<0.01 compared with naive and/or non-conditioned naive mice. Error bars indicate S.E.M.

In the Pavlovian fear conditioning task, under a weak training protocol there were no significant pre-tone differences among earthquake-experienced, naive, and non-conditioned naive mice 1 h after conditioning. However, earthquake-experienced mice displayed significantly greater conditioned freezing upon tone presentation (Figure 4A). A mixed design two-way ANOVA (with group as the between-subject factor and tone presentation as the within-subject factor) revealed significant main effects of group (*F* (2, 20) = 4.45, *p*<0.05), tone presentation (*F* (1, 20) = 58.61, *p*<0.001), and interaction (*F* (2, 20) = 5.76, *p*<0.05). Tukey-Kramer multiple comparison tests revealed a significant difference between earthquake-experienced and non-conditioned naive mice (*p*<0.05), but no difference between naive and non-conditioned naive or between earthquake-experienced and naive mice.

With respect to tone-dependent long-term memory, which was examined 24 h after conditioning (Figure 4B), two-way ANOVA revealed significant main effects of group (*F* (2, 20) = 12.58, *p*<0.001), tone presentation (*F* (1, 20) = 72.18, *p*<0.001), and interaction (*F* (2, 20) = 8.34, *p*<0.01). Tukey-Kramer post-hoc comparison revealed significant differences between earthquake-experienced mice and the two groups of naive mice (*p*<0.01). Although similar enhancement in conditioned freezing of earthquake-experienced mice was observed in the context-dependent test 48 h after conditioning (Figure 4C), group differences were marginally significant (one-way ANOVA: *F* (2, 20) = 3.11, *p* = 0.067). No significant differences were observed between earthquake-experienced and naive mice in conditioned freezing under the strong training protocol (Table 3).

**Table 3:** Fear conditioning task under strong training protocol. *

	Short-term		Long-term		
	Pre-tone	Tone	Pre-tone	Tone	Long-term context
Naive	10.8±2.6	33.2±5.5	9.3±2.6	42.0±7.2	42.3±7.4
Earthquake-experienced	17.1±4.0	42.2±3.9	16.6±3.9	45.1±5.9	54.7±5.8

* Mean freezing percentage of earthquake-experienced (n = 13) and naive mice (n = 9) in Pavlovian fear conditioning under a strong training protocol. Values are averages ± S.E.M.

In the weak training protocol, no significant differences were observed between naive and non-conditioned mice throughout the experiment (Figure 4); thus, it is reasonable to consider that the weak training protocol used in this study was too weak to induce conditioned fear in these animals. Earthquake-experienced mice, however, exhibited significant conditioned fear throughout the experiment, under protocol that did not induce fear in naive and non-conditioned mice. These results suggest that earthquake-experienced mice are considerably better at associating two stimuli (electric shock and tone or context), and thereby acquire conditioned fear under weak training protocol even though these protocol usually do not induce conditioned fear in naive animals. Similar conditioned fear was obtained in two groups of mice with the strong training protocol (Table 3), indicating that the strong training protocol saturates freezing behavior in all groups of mice (i.e., ceiling effect).

In the hotplate test, latency to lick paw was 6.8±1.5 s for earthquake-experienced mice and 6.7±1.5 s for naive mice. No differences in paw-lick latency were found between earthquake-experienced and naive mice (*t* (43) = 0.17, *n.s.*). Although the type of stimuli used in the fear conditioning task and hotplate test is different, both are associated with pain sensations. Thus, the hotplate test results suggest that enhanced freezing of earthquake-experienced mice in the fear conditioning task is not due to differences in pain sensitivity.

Mean serum corticosterone level was 114.8±38.9 ng/ml for earthquake-experienced mice and 64.6±20.6 ng/ml for naive mice. A t-test showed that the mean corticosterone concentration of earthquake-experienced mice was significantly higher than that of naive mice (*t* (17) = 3.66, *p*<0.01).

## Discussion

In the present study, we demonstrated that the earthquake that hit Japan on March 11, 2011 and long-lasting aftershocks drastically influenced the behaviors of mice. Earthquake-experienced mice displayed various behavioral alterations, including increased food consumption without body-weight gain, lower locomotion, higher level of anxiety, altered acquisition in water maze task, and enhanced memory to electric shock (Figures 2, 3, 4). These drastic behavioral changes after the earthquake were not well documented. A clue to explaining these earthquake-related behavioral changes can be found in previous studies that focused on stress-related behavioral changes in rodents [15]–[21]. Stress elicits a variety of behavioral changes, including lower locomotion and higher levels of anxiety [20], and increased food consumption [21], all similar to that observed in the earthquake-experienced mice in our study (Figure 2A). Increased food consumption without body-weight gain also was observed in rats before the 2008 Wenchuan earthquake in China [22]. In these rats, glucose uptake in skeletal muscle and adipose tissue also were reduced [22]. Therefore, earthquakes may alter metabolic states such that body weight remains relatively constant in spite of increased food consumption. Further examination is required in order to determine the detailed mechanisms underlying these metabolic changes.

The effect of stress on cognitive function remains controversial [23]. Stress-induced cognitive enhancement has been reported in rats exposed to unpredictable stress for 10 days, followed by trained in the Morris water maze task [18]. They reported that the escape latency of stressed rats was significantly shorter than that of control rats in the water maze task [18]. These results are very similar to our present results (Figure 3). Since adrenalectomy damages the hippocampal dentate gyrus and impairs Morris water maze performance [24], and since corticosterone administration or moderate stress improves water maze performance [15], Gouirand and Matuszewich (2005) concluded that repeated exposure to stress increases corticosterone concentrations to levels that enhance task acquisition. In another study that focused on the role of stress on memory processes, the arousal of emotions caused by a stressful event led to the activation of the amygdala, which then improved memory consolidation in several brain regions, including the hippocampus, through the activation of glucocorticoid receptors [25].

Since electrical foot shocks often are used as stressors in a variety of paradigms [16], [26], it is possible that the electrical foot shocks used in our fear conditioning task might have served as a stressful event causing emotional arousal. We, however, observed that earthquake-experienced and naive mice had comparable pain sensitivity in the hotplate test. Thus, it is reasonable to assume that pain-induced arousal of emotions and subsequent memory consolidation are equivalent in earthquake-experienced and naive mice. It has been reported that the combination of dietary restriction and stress exposure resulted in over-eating, which did not occur by either dietary restriction or stress alone [16]. Along with elevated corticosterone levels, increased food consumption in the earthquake-experienced mice suggest that repeated and continued aftershocks may serve as sufficient stressors to elicit several behavioral changes in these mice. Since strong correlation has been reported between elevated corticosterone and eating behavior [27]–[29], elevated corticosterone in earthquake-experienced mice might account for their increased food consumption (Figure 2A). Though there was a clear difference in serum corticosterone concentration between earthquake-experienced and naive mice, further confirmation using the mice group without the behavioral test battery is necessary to establish that the serum corticosterone was indeed elevated only by earthquake experience because the mice used for the corticosterone measurement have undergone behavioral test battery.

In our study, we intentionally started the behavioral experiments approximately one month after the main seismic event in order to see whether the behavioral changes had subsided. Although earthquakes are indeed potential stressors, it is unclear whether the behavioral changes we observed in our mice were caused by the main seismic event of March 11, or by the repeated aftershocks in addition to the main seismic event because minor aftershocks still continued during the testing period for the earthquake-experienced mice (Fig 1B). Moreover, how the intensity and the frequency of earthquakes influence animal behavior remains unknown. Since vibrations have been used as stressors in previous studies [17]–[19], the vibrations caused by an earthquake can be assumed to be the primary stressor associated with an earthquake. Although the mice experienced vibrations routinely during bedding exchange, they might have experienced the vibrations due to earthquakes much differently. Dietary restriction-induced stress should also be taken into consideration, as dietary restrictions are also used as a stressor [17]–[19]. Dietary restriction schedule and its duration were the same for the earthquake-experienced and naive mice used in the Morris water maze task. Although it is hard to assume that dietary restriction-induced stress directly and independently affect on the acquisition of spatial memory, we cannot exclude possible interactions between dietary restriction and seismic events on earthquake-related behavioral changes, including water maze performance and food consumption. Differences in the water maze performance in early stage of training might be explained by the different motivational state because swim speed is correlated with the increased motivation in the Morris water maze task [30]. It is, however, difficult to manipulate or quantify the extent of motivation using water maze task. Thus, the effect of motivational state in water maze cannot be dissociated from processing ability of spatial cognition *per se*. Because the asymptotic rate of decrease in escape latency and the probe test performance were similar for naive and earthquake-experienced mice, the altered performance of earthquake-experienced mice might be interpreted that it reflected an accelerated spatial memory acquisition, not an improved basal cognitive ability.

In the natural environment, animals have to perceive the subtle environmental changes such as water quality for their survival. Alteration of dissolved organic compounds in the ground water is hypothesized to be one of seismic precursors in the wild animals [4]. Though we could not find any major changes in water quality (Table 2), further experiments is necessary to clarify the effect of undetected organic compounds on the behaviors of the mice kept under a stable and well-controlled environment. In addition, subtle differences in the local environment may affect rodent behavior [31]–[33], other facility that experienced the same earthquake and aftershocks might have observed different behavioral change in mice. There is also a possibility that the mouse behaviors were influenced by magnetic field alterations associated with the earthquake [5]. Further examination is needed to reveal the effect of these environmental factors on laboratory mice behaviors.

A variety of experiences or events [34]–[36], such as natural disasters like earthquakes [37], [38], can cause posttraumatic stress disorder (PTSD) in humans. Thus, in the present study, the behavioral changes we observed in the earthquake-experienced mice in fact might be high-stress- and PTSD-like symptoms. Recent studies demonstrated that general population experienced the 2011 Tohoku earthquake expressed higher anxiety, changes in emotional states, and PTSD symptoms [39], [40] along with elevated cortisol level [39], which showed similar symptoms to the earthquake-experienced mice in our study. In addition, MRI study demonstrated the brain structural changes in human with PTSD symptoms caused by Tohoku earthquake [41]. It is indispensable to understand the neurological mechanism and epidemiology of human PTSD for the therapeutic purpose, on the other hand, it is also argued that investigation or research using traumatized humans are accompanied by ethical difficulties [42]. Therefore, earthquake-experienced animals might serve as an appropriate model to avoid the ethical concerns. Further examination is necessary to establish whether the behavioral alterations we observed in earthquake-experience mice were indeed PTSD-like symptoms.

The results of the present study provide us with an important lesson: Researchers should carefully monitor mice and observe and document their behaviors for several months after an earthquake. Nevertheless it has been reported that the housing environment affects behavior and physiology of laboratory animals [43], researchers usually do not determine whether the behaviors of their laboratory animals are affected by unexpected external factors [44], as we have reported here. Earthquake engineering such as seismic base isolation or aseismatic structures are widely introduced to reduce the risk for the destruction of vivarium or building, however, such techniques are unable to completely diminish or neutralize the transmission of ground-borne vibration elicited by intensive earthquake. Mice are widely used in scientific research around the world, and earthquakes can hit anywhere in the world. Furthermore, genetic modifications, such as gene deletion or overexpression, may potentially make these mice prone to the physiological and behavioral consequences of earthquakes. Thus, researchers must cautiously interpret any results obtained from mice exposed to earthquakes. The effects an earthquake may have on mouse behavior may not be readily noticeable and may last longer than we think.
